# Delayed rhabdomyolysis with paclitaxel, ifosfamide, carboplatin, and etoposide regimen: a case report

**DOI:** 10.1186/s13256-017-1272-9

**Published:** 2017-04-11

**Authors:** Alexandra Sokolova, Onyee Chan, Waqas Ullah, Auon Abbas Hamdani, Faiz Anwer

**Affiliations:** 1 Department of Medicine, Nassau University Medical Cite Center, 2201 Hempstead Turnpike, East Meadow, NY 11554 USA; 2 Department of Medicine, University of Arizona, Banner University Medical Center – Tucson, 1501 N. Campbell Ave., Tucson, AZ 85724 USA; 3 Department of Hematology, Oncology, Blood & Marrow Transplantation, University of Arizona, Banner University Medical Center – Tucson, 1501 N. Campbell Ave., Tucson, AZ 85724 USA

**Keywords:** Rhabdomyolysis, Testicular cancer, TI-CE chemotherapy, Carboplatin, Etoposide

## Abstract

**Background:**

High-dose chemotherapy with autologous stem cell rescue is commonly used for the treatment of relapsed germ cell tumors. We report the first case of delayed rhabdomyolysis with paclitaxel, ifosfamide, carboplatin, and etoposide regimen.

**Case presentation:**

We report a case of a 21-year-old African-American man diagnosed with relapsed non-seminomatous germ cell tumor who received high-dose chemotherapy with carboplatin and etoposide following TIGER trial arm B off-protocol. His course was complicated by muscle pain and rhabdomyolysis after cycle 4 on day +12 after infusion of autologous stem cells. To the best of our knowledge, this complication has not been reported with this regimen. A differential diagnosis of sepsis and neutropenic fever along with side effects of high-dose chemotherapy were considered, but based on the timing of events, it was concluded that the etiology of rhabdomyolysis is high-dose chemotherapy. Rhabdomyolysis was successfully treated with hydration and did not recur during subsequent cycle 5.

**Conclusions:**

Delayed rhabdomyolysis after high-dose chemotherapy with paclitaxel, ifosfamide, carboplatin, and etoposide regimen has not been previously reported and needs to be considered for preventive strategy and prompt diagnosis and treatment to avoid renal complications. Physicians should have a low threshold to check creatine kinase enzymes in patients with unexplained muscle pain or renal insufficiency after high-dose chemotherapy.

## Background

Germ cell tumor (GCT) even with metastatic disease responds well to initial chemotherapy. For patients who are appropriate for chemotherapy by staging, a cisplatin-based regimen such as cisplatin, bleomycin, and etoposide (BEP) is commonly used [1]. Around 10% of the patients relapse after first complete remission [2].

There has been controversy surrounding the use of conventional-dose chemotherapy (CDT) versus high-dose chemotherapy (HDCT) with autologous stem cell rescue as initial salvage therapy for relapsed GCT. TIGER is a clinical trial comparing HDCT and CDT for refractory GCT treatment. The TIGER trial used the paclitaxel, ifosfamide, carboplatin, and etoposide (TI-CE) regimen for the HDCT arm: paclitaxel and ifosfamide (TI) as initial salvage chemotherapy along with autologous stem cell collection with granulocyte-colony stimulating factor (GCSF) support followed by carboplatin and etoposide (CE) [3]. The high-dose component of the therapy consists of three cycles of CE. Each cycle of CE is supported with stem cell infusion as autologous rescue. Table 1 compares Einhorn regimen [4] to TIGER (TI-CE) regimen. In TI-CE approach, CE high-dose therapy is given in three cycles every 21 days, whereas under the Einhorn regimen, it is given in two cycles.

**Table 1:** Comparison between paclitaxel, ifosfamide, carboplatin, and etoposide regimen and Einhorn regimen

	TI-CE regimen	Einhorn regimen
Conventional chemotherapy TI = 2 cycles Einhorn cytoreduction 1–2 cycles	Paclitaxel (T) Ifosfamide (I)	Ifosfamide Cisplatin Vinblastine
Number of high-dose cycles with stem cell rescue	3	2
Etoposide dose	400 mg/m^2^ (D 1–3)	750 mg/m^2^ (D 1–3)
Carboplatin dose	AUC = 8 (D 1–3)	700 mg/m2 (D 1–3)

Einhorn regimen uses carboplatin 700 mg/m^2^ body-surface area instead of AUC = 8 and etoposide 750 mg/m^2^, which is higher than the dose in TIGER regimen (etoposide 400 mg/m^2^). In TI-CE approach, carboplatin and etoposide high-dose therapy is given in three cycles every 21 days, whereas in Einhorn regimen, it is given in two cycles

*AUC* area under the concentration-time curve, *D* days, *TI* paclitaxel and ifosfamide, *TI-CE* paclitaxel + ifosfamide and carboplatin + etoposide

Rhabdomyolysis is a rare complication of HDCT for testicular cancer [5]. We present a case of early relapse metastatic testicular cancer treated with HDCT TI-CE regimen complicated by rhabdomyolysis during cycle 4. This unusual adverse side effect has not been described in the literature using this regimen. It is important to keep in mind that rhabdomyolysis can be a possible complication in patients receiving HDCT with curative intent with autologous rescue for GCT in order to prevent renal failure.

## Case presentation

**Fig. 1:**
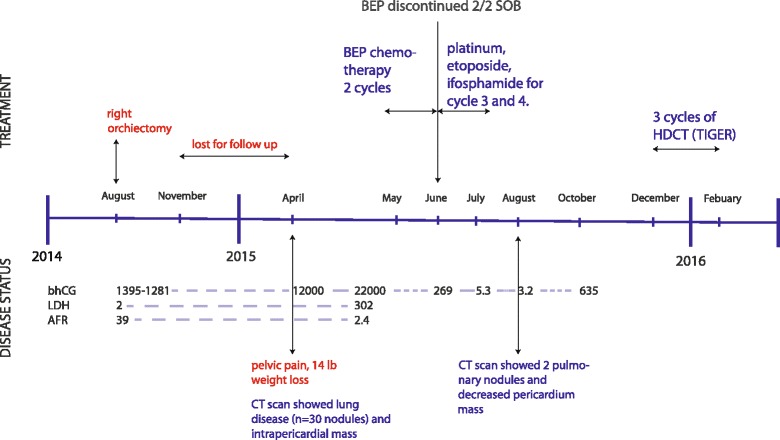
Disease timeline. In summary, our patient received two cycles of cisplatin, bleomycin, and etoposide and then platinum, etoposide, and ifosfamide for two cycles with some dose reduction of ifosfamide in cycle 4. His beta-human chorionic gonadotropin was 5.3 IU/L after four cycles of cisplatin, bleomycin, and etoposide/platinum, etoposide, and ifosfamide treatment. A computed tomography scan done 1 month after completing chemotherapy demonstrated significant improvement and only a few subcentimeter pulmonary nodules along with necrotic lymph node within his pericardium. Post-therapy, his beta-human chorionic gonadotropin further decreased to 3.2 IU/L. On a follow-up visit 4 months after chemotherapy, his beta-human chorionic gonadotropin went up to 635 IU/L. He was considered for salvage with TIGER trial-based paclitaxel, ifosfamide, carboplatin, and etoposide regimen. *AFR* alpha-fetoprotein, *BEP* cisplatin, bleomycin, and etoposide, *bhCG* beta-human chorionic gonadotropin, *CT* computed tomography, *HDCT* high-dose chemotherapy, *LDH* lactate dehydrogenase, *SOB* shortness of breath

We present the case of a 21-year-old African-American man who presented to his primary care physician with several months history of right testicular swelling. He underwent orchiectomy and his histopathology report was positive for a 2.5 cm mass, with 100% embryonal cells with lymphovascular invasion. The tumor was localized to his testis and epididymis and therefore staged as T1 disease. His pre-surgical baseline alpha-fetoprotein value was 39 ng/mL and beta-human chorionic gonadotropin (beta-hCG) level was 1395 IU/L. Surveillance was chosen as the strategy after orchiectomy. Unfortunately, close follow-up was not available because he moved out of the area. Approximately 8 months later, he developed pelvic pain and reported 14 pounds (6.3 kg) weight loss. His beta-hCG increased to 12,000 IU/L and a subsequent computed tomography (CT) scan of his chest/abdomen/pelvis revealed metastatic disease consistent with relapse of testicular cancer. There were up to 30 lung nodules and a large left-sided intra-pericardial mass (6×7×8 cm). We thought he had an intermediate risk disease and he received two cycles of three-drug combination BEP. He showed partial response with decrease of beta-hCG to 269 IU/L within a month. However, BEP had to be discontinued due to shortness of breath probably secondary to bleomycin-related lung damage. He later received platinum, etoposide, and ifosfamide (VIP) for cycle 3 and cycle 4. He developed confusion and erratic behavior, which was thought to be due to ifosfamide-related central nervous system (CNS) toxicity, and its dose was reduced in cycle 4. In summary, he received two cycles of BEP and then VIP for two cycles with some dose reduction of ifosfamide in cycle 4. His beta-hCG was 5.3 IU/L after four cycles of BEP/VIP treatment. A CT scan done 1 month after completing chemotherapy demonstrated significant improvement and only a few subcentimeter pulmonary nodules along with necrotic lymph node within his pericardium. Post-therapy, his beta-hCG further decreased to 3.2 IU/L. On a follow-up visit 4 months after chemotherapy, his beta-hCG went up to 635 IU/L. He was considered for salvage with TIGER trial-based TI-CE regimen (Fig. 1).

TI-CE consists of an initial salvage with two 14-day cycles of TI followed by stem cell collection and three cycles of CE, each supported with stem cell infusion [3]. The dosing protocol is summarized in Table 2.

**Table 2:** Summary of paclitaxel, ifosfamide, carboplatin, and etoposide therapy cycles given to our patient

Cycles	Frequency	Days
1–2	Every 14 days	D 1: paclitaxel 250 mg/m^2^ IV over 24 hours D 2–4: ifosfamide 2000 mg/m^2^ IV + mesna support D 4–14: GCSF 10 mcg/kg per day D 11–14: stem cell collection (28×10^6^/kg CD34+ cells collected)
3–5	Every 21 days	D 1–3: carboplatin AUC = 8 IV + etoposide 400 mg/m^2^ IV D 5: stem cell infusion (9.65×10^6^/kg CD34+ cells with each cycle) D 3–21: GCSF 10mcg/kg per day

*AUC* area under the concentration-time curve, *D* days, *GCSF* granulocyte-colony stimulating factor, *IV* intravenous

He tolerated the first cycle of CE-based HDCT (regimen cycle 3) without much difficulty except for culture-negative neutropenic fever despite prophylactic acyclovir 800 mg by mouth twice a day, fluconazole 200 mg by mouth daily, and ciprofloxacin 500 mg by mouth twice a day. Vancomycin 1250 mg administered intravenously twice a day and cefepime 2 gm administered intravenously daily were administered empirically for neutropenic fever. His neutropenic fever resolved and he was discharged on day +12.

**Fig. 2:**
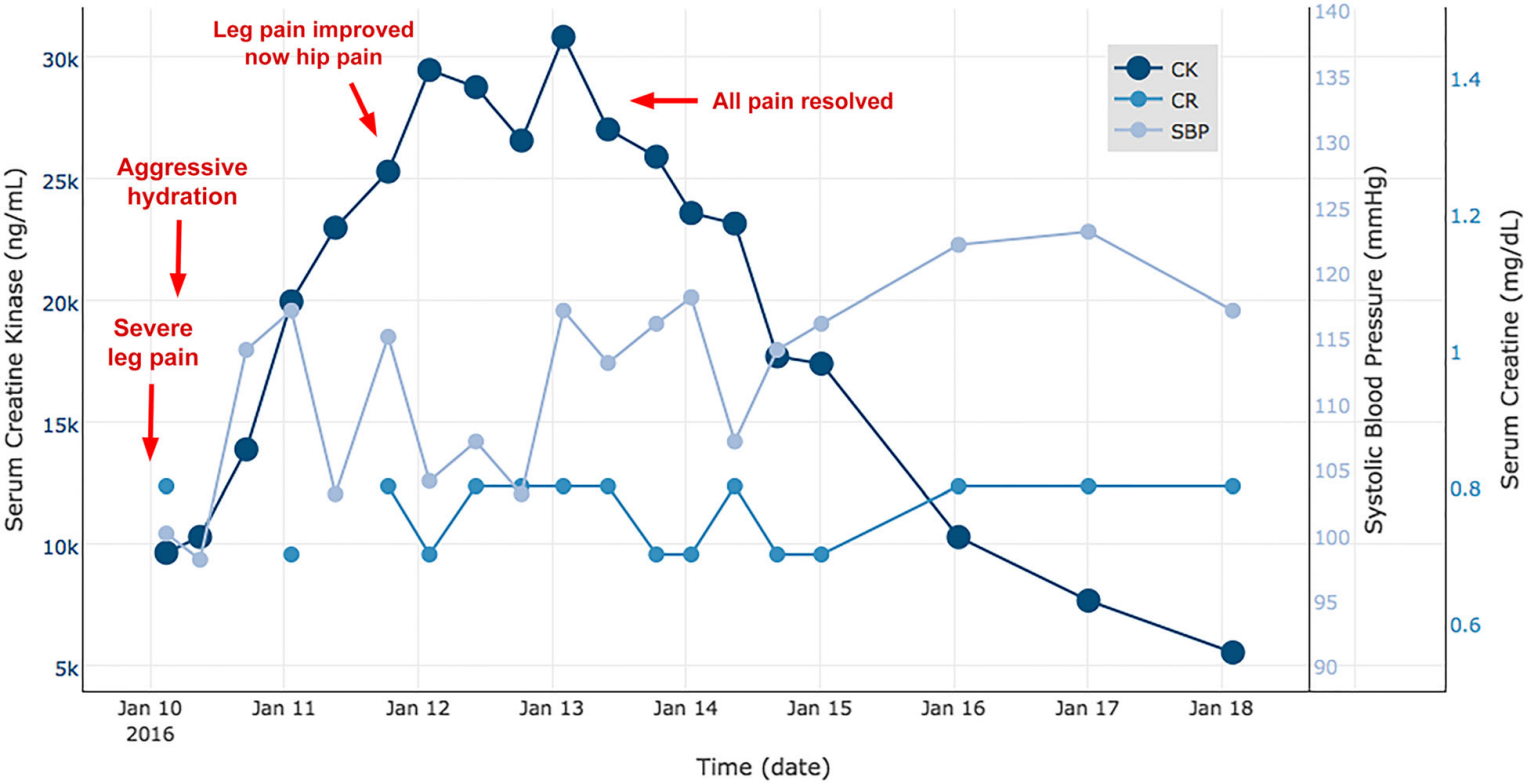
Creatine kinase levels. Our patient developed leg and hip pain as rhabdomyolysis started to develop. Creatine kinase peaked at above 30,841 ng/mL. His systolic blood pressure was close to his baseline throughout his hospitalization, which indicates hypoperfusion as the etiology of his rhabdomyolysis is unlikely. As a result of prompt aggressive hydration, our patient did not develop renal failure and his rhabdomyolysis completely resolved. *CK* creatine kinase, *CR* creatine, *SBP* systolic blood pressure

A second cycle of HDCT (regimen cycle 4) was complicated by severe sepsis due to *Streptococcus mitis* bacteremia on day +9. He was transferred to our intensive care unit and required vasopressor support for septic shock for a few hours. The septic shock improved within 48 hours with fluids and vasopressor support. A transesophageal echocardiogram was negative and not suggestive of vegetation. He was successfully treated with antibiotics administered intravenously: meropenem 500 mg administered intravenously every 6 hours, vancomycin 1250 mg administered intravenously twice a day, and cefepime 2 gm administered intravenously every 8 hours. On day +12 he developed bilateral leg pain and was diagnosed with rhabdomyolysis when his initial creatine kinase (CK) level was found to be elevated at 9673 IU/L. His CK was monitored serially and started trending up and reached a peak value of 30,841 IU/L on day +16. He was promptly treated with fluids administered intravenously and his CK values started to improve (Fig. 2). His cardiac enzymes were within normal limits. His hypotension due to shock resolved more than 24 hours before symptoms of leg pain and elevation in CK. Rhabdomyolysis was thought to be secondary to HDCT as sepsis and hypotension had already resolved with antibiotics, supportive therapy, and fluids.

Finally, regimen cycle 5 was tolerated without major complications. He developed diarrhea with stool positive for *Clostridium difficile* and was treated with oral metronidazole 500 mg every 8 hours for 10 days. He underwent restaging 3 months after completing HDCT and had no evidence of active malignancy. He continues to do well 4 months after completion of therapy and will remain in close follow-up.

## Discussion

The TIGER trial is a currently ongoing phase III trial designed to compare CDT (arm A, paclitaxel, ifosfamide, and cisplatin, TIP, × 4) and HDCT (arm B using TI-CE) as initial salvage treatment in relapsed and refractory GCTs.  The result of this trial is currently pending [3]. Development of stem cell autologous rescue after HDCT has decreased mortality from 20% to 2 to 3% [6]. Nevertheless, HDCT still has major acute and chronic regimen-related toxicities (RRT). End organ damage depends on the number of cycles, time period between cycles, and specific chemotherapy drugs used in addition to underlying comorbidities. Common side effects of HDCT include bone marrow suppression (neutropenia, thrombocytopenia, and anemia), nausea, vomiting, fatigue, mucositis, and diarrhea. HDCT can also lead to secondary solid tumors or hematologic malignancies such as leukemias [7]. Carboplatin-based therapy is also known to cause peripheral neuropathy and hearing loss.

Rhabdomyolysis is one of the established side effects of HDCT. Rhabdomyolysis is diagnosed when CK levels are five times above the normal range with no accompanying elevation of cardiac or brain fraction. It can present with muscle pain, weakness, vomiting, and confusion. Kidney damage by myoglobin leads to acute kidney injury (AKI). Rhabdomyolysis can be caused by exercise, crush injuries, muscle ischemia, hypothermia, hyperthermia, and by use of some drugs such as statins commonly used for lipid disorders. Some chemotherapy drugs are known to cause rhabdomyolysis and they include: ifosfamide, CE [5], trabectedin [8], gemcitabine/paclitaxel [9], cytarabine [10], doxorubicin, thioguanine, vincristine [11], mitoxantrone/cyclophosphamide [12], pemetrexed [13], high-dose cyclophosphamide (120 mg/kg) [14], and 5-azacytidine.

A patient with rhabdomyolysis should have close clinical monitoring including monitoring for serum creatinine and urine output monitoring because of the risk of developing oliguric AKI. Serial CK values are required to evaluate the response to treatment. Close electrolyte monitoring is needed as patients can develop hyperkalemia (in that case cardiac monitoring might be required) and low calcium. Patients are at risk of developing complications such as compartment syndrome and disseminated intravascular coagulation. The main treatment for rhabdomyolysis is aggressive hydration administered intravenously (6 to 12 liters over 24 hours). Bicarbonate if indicated for acidosis should be used with caution in patients with low calcium and high phosphate as it can precipitate calcium phosphate deposition. In severe cases, patients may require hemodialysis.

The exact mechanism of how chemotherapy agents cause rhabdomyolysis is variable and not always clear. Ifosfamide is thought to cause heart muscle damage more than skeletal muscular damage [15]. Rhabdomyolysis usually develops on days 1 to 3 of chemotherapy [16]. In our patient, rhabdomyolysis developed on day 13 of regimen cycle 4. He also had sepsis during this cycle, which could have contributed to the development of rhabdomyolysis. However, no other obvious causes of rhabdomyolysis (e.g. trauma, alcohol, seizures, myositis, ischemia) were identified. Without a high index of suspicion, serum CK levels are not checked during routine care of patients during HDCT/autologous rescue. Musculoskeletal aches and pains are common during HDCT regimens as patients are routinely getting GCSF to facilitate engraftment, hence rhabdomyolysis can go unrecognized resulting in severe renal injury. Delayed rhabdomyolysis with TI-CE regimen has not been reported in the literature.

## Conclusions

Rhabdomyolysis is a recognized side effect of high-dose chemotherapy. A newly recognized side effect of delayed rhabdomyolysis with symptoms of bilateral leg muscle pain was documented in this case with TI-CE regimen. HDCT-induced rhabdomyolysis responded to aggressive hydration administered intravenously in our case. To prevent rhabdomyolysis as a complication of HDCT and promptly address it in a timely manner on presentation, physicians should have a low threshold to check CK levels in patients with unexplained muscle pain or new onset of renal insufficiency.

## Abbreviations

AKI: Acute kidney injury
BEP: Chemotherapy regimen, including cisplatin, bleomycin, and etoposide
CDT: Conventional-dose chemotherapy
CE: Carboplatin and etoposide
CK: Creatine kinase
CNS: Central nervous system
CT: Computed tomography
GCSF: Granulocyte-colony stimulating factor
GCT: Germ cell tumor
HCG: Human chorionic gonadotropin
HDCT: High-dose chemotherapy
RRT: Regimen-related toxicities
TI: Paclitaxel and ifosfamide
TI-CE: Chemotherapy regimen, including paclitaxel (T), ifosfamide (I) carboplatin (C), etoposide (E)
TIP: Chemotherapy regimen, including paclitaxel (T), ifosfamide (I), cisplatin (C)
VIP: Chemotherapy regimen, including platinum, etoposide and ifosfamide
